# Lipid profile after omega-3 supplementation in neonates with intrauterine growth retardation: a randomized controlled trial

**DOI:** 10.1038/s41390-023-02632-z

**Published:** 2023-05-18

**Authors:** Mai Elsheikh, Doaa El Amrousy, Heba El-Mahdy, Heba Dawoud, Ahmed Harkan, Amany El-Barky

**Affiliations:** Pediatric Department, Tanta University, Tanta, Egypt

## Abstract

**Background:**

Neonates with intrauterine growth restriction (IUGR) have a high lipid profile that predisposes them to cardiovascular disease later in life. We aimed to evaluate the effect of omega 3 supplementation on serum leptin level, lipid profile, and growth in neonates with IUGR.

**Methods:**

This clinical trial was conducted on 70 full-term neonates with IUGR. Neonates were randomly divided into two equal groups; the treatment group: received omega 3 supplement (40 mg/kg/day) for 2 weeks after the establishment of full feeding, and the control group, who were followed up to full feeding without any supplementation. Serum leptin level, total cholesterol (TC), high-density lipoprotein (HDL), triglycerides (TG), low-density lipoprotein (LDL), and anthropometric measurement were evaluated at admission and after 2 weeks of omega 3 supplementation in both groups.

**Results:**

After treatment, HDL significantly increased, unlike TC, TG, LDL, LDL, and serum leptin levels, which significantly decreased in the treatment group compared to the control group after treatment. Interestingly, weight, length, and ponderal index greatly increased in omega 3-treated neonates compared to the control group.

**Conclusion:**

Omega 3 supplementations lowered serum leptin level, TG, TC, LDL, and VLDL but increased HDL and growth in neonates with IUGR.

**Clinical trial registration:**

The study was registered at clinicaltrials.gov (NCT05242107).

**Impact:**

- Neonates with intrauterine growth retardation (IUGR) were reported to have a high lipid profile that predisposes them to cardiovascular disease later in life. Leptin is a hormone that adjusts dietary intake and body mass and has a significant role in fetal development. Omega 3 is known to be essential for neonatal growth and brain development.
- We aimed to evaluate the effect of omega 3 supplementation on serum leptin level, lipid profile, and growth in neonates with IUGR.
- We found that omega 3 supplementations lowered serum leptin level and serum lipid profile but increased high density lipoprotein and growth in neonates with IUGR.

## Introduction

Intrauterine growth restriction (IUGR) is a significant public health problem with significant perinatal mortality and morbidity.^1^ IUGR is the first hit that adversely affects the fetus’s developmental programming. This early misprogramming increases the susceptibility to cardiovascular diseases, coronary heart disease, hypertension, and type 2 diabetes mellitus.^2^

Neonates with IUGR were reported to have abnormal lipid profiles. High triglycerides (TG) and low high density lipoprotein (HDL) levels in neonates with IUGR may be due to enhanced cholesteryl ester transfer, which could explain why neonates with IUGR have a higher risk of coronary heart disease later in life.^3^

Leptin adjusts dietary intake, body mass, and has a significant role in the fetal development, angiogenesis, and lipolysis. Trials reported that the level of serum leptin is reduced during fasting or energy restriction and increased during re-feeding, overfeeding, and surgical stress.^3^ While excess leptin provides energy expenditure by suppressing food intake and increasing thermogenesis, decreased leptin increases the storage in adipocytes. Several studies reported that plasma leptin levels were positively correlated with body mass index (BMI) values and total body fat.^4–6^ Also, Marchini et al.^5^ found that the plasma leptin level decreases in connection with the initial physiological weight loss in newborn infants. Following this discovery,^7^ leptin and infant development have gained momentum.^8–10^

Understanding the leptin and cholesterol metabolism in the fetus and neonates is very critical, not only for the nourishment of neonates but also for the strategies to decrease the future recognized risk of cardiovascular diseases (CVD) in fetuses with IUGR.^2^

Higher intake of anti-inflammatory omega 3 polyunsaturated fatty acids (PUFA) in pregnancy has been associated with lower adiposity, higher birth weight and longer gestation.^11^ Omega 3 supplementation has multiple proposed mechanisms that could improve the metabolic adipocyte and lipid profile.^12^ These involve changes in the gene expression of adipose tissue, adipokine-mediated or related pathways, changes in adipokine release, alterations in the carbohydrate metabolism, appetite suppression, the increases in energy expenditure (possibly through thermogenesis), the increases in fat oxidation, activating mechanisms involved in muscle anabolism, and its effect on epigenetics.^13^ Omega 3 supplementation effect on leptin levels is still controversial; some studies on omega 3 supplementation reported that it decreases circulatory leptin levels^14–17^; however, other investigations do not support this beneficial effect of omega 3 intake impact on leptin concentrations.^18–20^

There is a paucity of trials that focus mainly on the omega 3 effect on leptin serum level in neonates with IUGR. Therefore, our study aimed to assess the effect of omega 3 supplementation on serum leptin level and lipid profile in neonates with IUGR.

## Material and methods

This randomized open-label clinical trial was carried out on 70 full-term neonates with IUGR who were referred to the neonatal intensive care unit (NICU), Pediatric Department, Tanta University Hospitals from June 2021 to January 2022. The trial was done after approval from the Ethical Committee of the Faculty of Medicine at Tanta University (code:35199/1/22). The trial was registered at clinicaltrials.gov (ID: NCT05242107). The guardians of included neonates provided informed consent.

Exclusion criteria were preterm neonates, neonates who died before feeding was established, neonates with congenital infection, neonates with respiratory distress, neonates with cholestasis, and neonates who had multiple congenital anomalies.

### Intervention

We used omega 300 (Montana pharmaceutical industry) capsule, which contains 1000 mg Omega 3 fatty acids 3 in a dose of 40 mg/kg/day in the study group.^19^ After the establishment of full-feeding and for 2 weeks duration, mothers learned how to use the capsule after calculating the suitable dose for their baby after recovery. The contents of the capsule were taken by a syringe, and the mother dissolved it in 10 ml of expressed breast milk or freshly prepared formula and then shook it very well; then the calculated dose was given to the baby once daily for 2 weeks.

### Randomization

Neonates who met the inclusion criteria were randomly assigned to one of two study groups in a 1:1 ratio using a random block size of six utilizing computer-generated random numbers by an independent statistician. Allocation concealment was accomplished using sequentially numbered sealed opaque envelopes. The sealed opaque envelope was opened once the patient’s guardians signed the written agreement and the neonates were enrolled into the appropriate group.

The treatment group: include 35 neonates who received omega 3 supplements (40 mg/kg/ day) after the establishment of full feeding for 2 weeks.

The control group: included 35 neonates who were followed up to full feeding without receiving any supplementation.

### Measurements

All patients were subjected to complete history taking such as maternal age, mode of delivery, and maternal dietary intake of omega 3 during pregnancy. Full clinical examinations were done for all included neonates e.g., gestational age assessment, vital signs, and anthropometric measurements such as weight, length, and head circumference (HC).

The Ponderal index (PI) can be used to detect newborns with soft tissue mass that is below average for their skeletal development stage. PI has been used to assess the IUGR because low birth weight and IUGR tends to reoccur in siblings, and clustering of PI in sibling even persists after controlling for factors such as race, sex, maternal age, gravidity, year of birth, gestational age, pregnancy complications and poor maternal illnesses.^21^

The formula for calculating the PI is as follows: PI = birth weight (g)/(length in cm)^3^ X 100. IUGR is diagnosed when PI is below 2. All measurements were taken at admission and after 2 weeks of omega 3 supplementation.

Routine laboratory investigations such as complete blood count, serum urea and creatinine, blood urea nitrogen, C- reactive protein, and serum electrolytes such as serum sodium (Na), potassium (K), and calcium (Ca) were also performed at admission and after 2 weeks from omega 3 supplementation.

Serum leptin level was measured using commercial ELISA assay kits (Ray Biotech Inc., Norcross).

Serum lipid profile included total cholesterol (TC), high density lipoproteins (HDL), triglycerides (TG), low density lipoprotein (LDL), and very-low-density lipoprotein (VLDL) were measured by enzymatic colorimetric method using commercial kits (Biodiagnostic, Dokki, Giza, Egypt).

All laboratory investigations were measured at admission and 2 weeks after omega 3 supplementation. Blood sampling was obtained before breastfeeding and at least 2 h after the previous feed between 8 and 10 am.

### Data collection and follow up

All cases were followed up as regards the establishment of full feeding, weight gain, length, and HC catch up of growth 2 weeks after completion of omega 3 supplementation. Development of signs and symptoms of sepsis, feeding intolerance, or necrotizing enterocolitis were observed. Moreover, any side effects of the drug were also recorded.

### The outcomes

The primary outcome was to assess the effect of omega 3 supplementation on serum leptin levels. The secondary outcomes were to assess the effect of the supplementation of omega 3 on serum lipid profile and on the anthropometric measurement.

### Sample size calculation

The sample size calculation was done by G*Power 3.1.9.2 (Universitat Kiel, Germany). We performed a pilot study on five cases in each group. The mean ± SD of leptin level after 2 weeks (the primary outcome) was 1.42 ± 0.148 ng/ml in the control group and 1.74 ± 0.52 in the study group. Four cases were added to each group to overcome dropout. We needed 35 cases in each group to achieve 90% power of the study with a 0.83 effect size and 0.05 α error.

The CONSORT 2010 checklist is included in the supplementary files (supplementary file 1).

### Statistical analysis

SPSS v27 (IBM, Chicago, IL) was used for the statistical analysis. Shapiro–Wilks test and histograms were used to determine the normality of the data distribution. The quantitative data were presented as mean and standard deviation (SD) if normally distributed, while non-parametric quantitative data were presented as the median and interquartile range (IQR). Qualitative variables were expressed as frequency and percentages (%). Normally distributed quantitative data were compared between the two groups using the unpaired Student *t*-test, while non-parametric quantitative data were compared using the Mann–Whitney test. Categorical data were compared between the two groups using Chi-square or Fisher’s exact tests. Paired *T*-test was performed to compare variable quantitative data within the same group. A two-tailed *P* ≤ 0.05 was considered statistically significant.

## Results

**Fig. 1:**
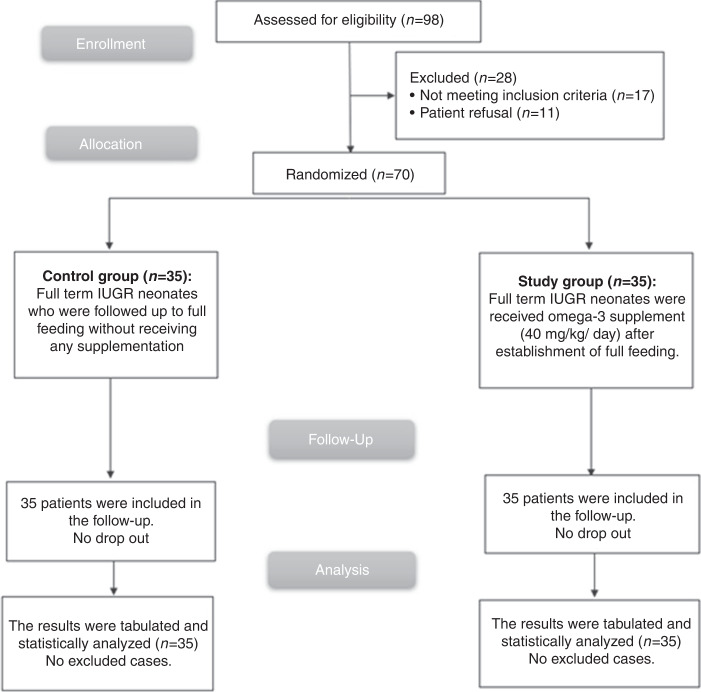
Consort flow diagram of the study. It showed participants through each stage of the clinical trial.

The flow chart of the present study was presented as Fig. 1.

There was an insignificant difference between both groups as regards postnatal age, gestational age, sex, mode of delivery, type of feeding, history of necrotizing enterocolitis (NEC), or maternal use of omega 3. Antenatal risk factors were significantly higher in the study group compared to the control group (*P* = 0.007) (Table 1).

**Table 1:** History and demographic data in the studied groups.

Parameters	Control group (*n* = 35)	Study group (*n* = 35)	*p*-value	
Postnatal age (hours)	1.5 ± 0.4	1.4 ± 0.6	0.214	
Gestational age (weeks)	38.4 ± 0.3	38.3 ± 0.4	0.381	
Sex	Male	19 (54.3%)	12 (34.3%)	0.092
	Female	16 (45.7%)	23 (65.7%)	
Modes of delivery	Vaginal	7 (20.0%)	10 (28.6%)	0.403
	CS	28 (80.0%)	25 (71.4%)	
Antenatal risk factor	Anemia	0 (0.00%)	9 (25.7%)	0.007*
	DM	16 (45.7%)	9 (25.7%)	
	HTN	17 (48.6%)	17 (48.6%)	
	Anemia + D.M + HTN	1 (2.9%)	0 (0%)	
Omega 3 mother. intake	0 (0.0%)	0 (0.0%)	–	
Types of feeding	Breast feeding	11 (31.4%)	12 (34.3%)	0.779
	Both	24 (68.6%)	23 (65.7%)	
Occurrence of NEC	0	0	–	

Data presented as mean ± SD or frequency (%).

*HTN* hypertension, *DM* Diabetes mellitus, *NEC* Necrotizing enterocolitis, *CS* caesarean section.

At admission, weight, length, head circumference, and ponderal index were insignificantly different between the two groups. Moreover, leptin, TC, TG, HDL, LDL, and VLDL were comparable between the two groups. No significant difference was found between both groups as regards CBC, renal function tests, serum electrolytes, or CRP (Table 2).

**Table 2:** Anthropometric and laboratory data in the studied groups at admission.

Parameters	Study group (*n* = 35)	Control group (*n* = 35)	*P*-value
Length (cm)	46.9 ± 2.3	46.2 ± 1.1	0.132
Birth weight (kg)	1.9 (1.70–1.95)	1.9 (1.8–2)	0.139
HC (cm)	32.1 ± 1.1	31.8 ± 0.7	0.172
Ponderal index	1.9 ± 0.3	1.9 ± 0.1	0.662
Hb (gm/dl)	15.5 ± 2.7	16 ± 1.9	0.905
Hct (%)	48.3 ± 5.7	49.2 ± 4.2	0.102
Platelet (10^3^/dl)	267.6 ± 86.1	248.9 ± 67.2	0.263
TLC (10^3^/dl)	9.0 [8.65–10.20]	8.5 (7.45–9.90)	0.451
Creatinine (mg/dl)	0.3 [0.25–0.30]	0.30 [0.20–0.40]	0.416
Urea (mg/dl)	24.7 ± 12.7	26.1 ± 5.4	0.566
Positive CRP (mg/L)	35 (100.0%)	33 (94.3%)	0.492
Na (mEq/L)	138 ± 5.8	139 ± 4.2	0.341
K (mEq/L)	3.9 ± 0.4	4 ± 0.5	0.278
Ca (mg/dl)	8.9 ± 0.6	9 ± 0.5	0.360
Leptin (ng/ml)	1.6 ± 0.4	1.8 ± 0.4	0.072
TC (mg/dl)	117.3 ± 28.5	105.9 ± 25.4	0.081
HDL (mg/dl)	16.1 ± 2.6	15.2 ± 3.5	0.241
TG (mg/dl)	66 (48–110)	75 (54–94)	0.461
LDL (mg/dl)	67 (55.50–88.50)	84 (64–96)	0.060
VLDL (mg/dl)	13 (9–21.5)	12 (10–18)	0.911

*Hct* hematocrit, *TLC* total leukocyte count, *Hb* hemoglobin, test, *Na* serum sodium, *K* serum potassium, *Ca* calcium, *CRP* C-reactive protein, *P < 0.05.

Leptin level significantly decreased after 2 weeks of omega 3 treatment in the study group compared to their levels at admission (*P* < 0.001). However, leptin significantly increased in the control group after treatment compared to their levels at admission (*P* < 0.001).

TC, TG, LDL, and VLDL significantly decreased after treatment in the study group compared to the control group (*P* ˂ 0.05), unlike HDL, which significantly increased after treatment in the study group compared to the control group (Table 3).

**Table 3:** Leptin and Lipid profile of the studied patients after treatment.

		At admission	After 2 weeks	*P* value
Leptin (ng/mL)	Control (*n* = 35)	1.8 ± 0.4	2.6 ± 0.5	<0.001*
	Study (*n* = 35)	1.7 ± 0.4	1.3 ± 0.3	0.02*
	*P*# value	NS	<0.001*	
TC (mg/dL)	Control (*n* = 35)	112.3 ± 26.05	111.5 ± 33.38	NS
	Study (*n* = 35)	111.97 ± 28.05	94.02 ± 26.16	<0.001*
	*P*# value	NS	0.017*	
HDL (mg/dL)	Control (*n* = 35)	15.2 ± 3.5	16.8 ± 4.5	NS
	Study (*n* = 35)	16.1 ± 2.6	25.9 ± 4.4	<0.001*
	*P*#-value	NS	<0.001*	
TG (mg/dL)	Control (*n* = 35)	75 [54–94.5]	71 [60.5–89.5]	NS
	Study (*n* = 35)	69 [48–110]	39.1 [28.6–69]	<0.001*
	*P*# value	NS	0.025*	
LDL (mg/dL)	Control (*n* = 35)	66 [55.5–79]	64 [52–77]	NS
	Study (*n* = 35)	72 [64–96]	50 [47–80]	<0.001*
	*P*# value	NS	<0.001*	
VLDL (mg/dL)	Control (*n* = 35)	12 [10–18]	13 [5.6–15]	NS
	Study (*n* = 35)	13 [9–21.5]	9 [7–14.5]	<0.001*
	*P*# value	0.911	0.001	

Data presented as mean ± SD or median [IQR].

*TC* total cholesterol, *HDL* high-density lipoprotein, *TG* triglycerides, *LDL* low-density lipoprotein, *VLDL* Very-low-density lipoprotein, *NS* not sigificant

*p < 0.05.

After treatment, weight and ponderal index significantly increased in both groups but more in the omega 3-treated group. Length significantly increased in the omega 3 treated group compared to the control group. However, HC was comparable in both groups after treatment. No remarkable side effects were recorded in our patients (Table 4).

**Table 4:** Anthropometric measurements of the studied patients after treatment.

		At admission	After 2 weeks	*P* value
Length (cm)	Control (*n* = 35)	46.20 ± 1.07	46.35 ± 1.05	NS
	Study (*n* = 35)	46.85 ± 2.31	49.02 ± 0.30	<0.001*
*P*# value		NS	0.001	
Head circumference (cm)	Control (*n* = 35)	31.77 ± 0. 73	33.01 ± 0.78	NS
	Study (*n* = 35)	32.08 ± 1.13	33.17 ± 1.12	NS
P# value		NS	NS	
Birth weight (kg)	Control (*n* = 35)	1.9 [1.80–2.00]	2.25 [2.10–2.28]	0.01*
	Study (*n* = 35)	1.9 [1.70–1.95]	3.10 [1.90–2.20]	<0.001*
P# value		NS	0.004*	
Ponderal index	Control (*n* = 35)	1.91 ± 0.08	2.1 ± 0.10	<0.001*
	Study (*n* = 35)	1.9 ± 0.3	2.7 ± 0.34	<0.001*
		NS	0.01	

Data presented as mean ± SD, or median [IQR].

*NS* not significant.

*p < 0.05.

## Discussion

Leptin, a 16 kDa protein hormone, induces a negative energy balance by elevating energy expenditure and decreasing food intake.^22^ Leptin may play a role in the control of substrate utilization and in the maintenance of fat mass before birth. Leptin synthesis and levels in the fetus are regulated and affected by several intrauterine and environmental circumstances, and it may signal the availability of nutrients and energy, particularly in the late stages of gestation. It is possible that in IUGR, the underlying mechanisms of inutero leptin action in the developing susceptibility to adult obesity are the alterations of the expression of appetite-stimulating neuropeptides such as NPY in the fetal brain.^23^ Adipocytokines, especially leptin, have been implicated in intrauterine growth, and unfriendly environments such as those related to IUGR may lead to upregulation in placental protein leptin synthesis as well as leptin mRNA in neonates with IUGR, as reported by ref. ^24^

In the current study, omega 3 significantly decreased leptin levels in neonates with IUGR compared to the control group in which leptin levels significantly increased after 2 weeks of treatment compared to their levels at admission. In line with our study, Hariri et al.^25^ performed a systematic review and meta-analysis and found that omega 3 supplementation might significantly reduce leptin concentration when compared with placebo in non-obese adults. Also, Gray et al.^17^ found that supplementation of omega 3 decreased plasma leptin levels in non-obese adults and conversely increased leptin levels in obese adults. It is reported that mean food intake over a few days or weeks is directly correlated with plasma leptin concentrations. A short-term adaptation to fasting may therefore be seen as a drop in plasma leptin levels. Leptin is believed to signify a set point at which energy intake and energy expenditure are equal in circumstances where weight and diet are consistent. This set point measures leptin sensitivity, which may vary depending on genetic, dietary, and/or environmental factors.^17^ There were few researches reporting on how omega-3 fatty acids affected leptin levels in human blood. Rausch et al.^26^ reported in a systematic review that eicosapentaenoic acid (EPA) and/or docosahexaenoic acid (DHA) supplementation decreased leptin levels that may be due to decreased inflammation through altering gene expression, reducing adipocyte size, decreasing weight and total adiposity, and improving insulin sensitivity.

Omega 3 fatty acids reduce the synthesis and increase the oxidation of fatty acid (FA).^27^ It has also been shown that n-3 FAs are ligands for peroxysomal proliferator-activated receptor α (PPARα) and PPARγ and can heterodimerize with retinoid X receptor to increase the expression of FA oxidizing enzymes and reduce the expression of sterol regulatory element-binding protein (SREBP)-1, promoting reduced lipogenesis.^28^ Hence, because omega 3 fatty acids seem to influence fatty acid metabolism markedly and leptin is important for adipose tissue size, omega 3 fatty acid supplementation exhibit a promising role in managing abnormal high leptin levels in IUGR.^29^

High lipid profile was reported in neonates with IUGR that indicate intrauterine malnutrition and lipolysis, and this is a key biochemical indicator of IUGR and contributes to a higher risk of metabolic and coronary heart diseases in the adult life.^30^ Metabolomic analysis of cord blood from early and late-onset IUGR revealed an abnormal lipid metabolism in both early and late-onset IUGR.^31^ Higher circulating triglycerides and cholesterol lipoproteins in growth-restricted fetuses were noted.^32^

In our study, we found that TC, as well as TG levels, significantly decreased in omega 3 treated neonates compared to the control group, which came in agreement with EL-alkamy et al.^33^ who investigated the effect of omega 3 on serum lipid profile level in 25 full-term neonates with IUGR compared to controls. They noted an increase in the level of TC and f TG initially in IUGR neonates, and this level was decreased after supplementation of Omega 3 for 2 weeks.

VLDL and IDL were found to be the most deregulated lipoproteins in fetuses with IUGR, suggesting that VLDL rich in triglycerides may be an alternative fuel mobilized by the growth-restricted fetus.^25^ We noted that LDL and VLDL significantly decreased after 2 weeks in the study group compared to the control group. In line with our results, EL-alkamy et al.^33^ found a significant decrease in LDL after 1 week and in VLDL after 2 weeks of Omega 3 supplementation in the cases group more than in the control group.

Also, Garaiova et al.^34^ reported in their study, which was done on 25 participants with a mean age of 16 years, that there was a significant decrease in levels of LDL after a daily supplementation of fish oil for 16 weeks (*p* < 0.05). Also, this came in line with Dallongeville et al.^35^ who reported a significant decrease in levels of VLDL in adult patients suffering from type 3 dys-beta-lipoproteinemia on receiving 6 grams of Omega 3 for 12 weeks.

Mechanistically, most evidence suggests that Omega 3 fatty acids reduce the synthesis and secretion of VLDL particles and increase TG removal from VLDL and chylomicron particles through the upregulation of enzymes, such as lipoprotein lipase.^36^

On the other hand, a low HDL level was noted in fetuese with IUGR when compared to appropriately grown fetuses. Although the cause of the low fetal HDL concentration in neonates with IUGR is still a matter of debate, it was suggested that HDL is the result of diminished fetal de novo synthesis.^37^ In the present study, HDL significantly increased in omega 3 treated neonates compared to the control group, which agreed with the results of El-alkamy et al.^33^ and Mohamed et al.^38^ studies. This witnessed effect might be attributed to that Omega 3 PUFAs, especially DHA, increase HDL-C.^39^ The formation of HDL is related to the catabolism of TG-rich lipoproteins such as VLDL or intermediate-density lipoprotein (IDL) by LPL.^40^ Therefore, increased LPL activity reduces IDL and VLDL and increases HDL. Increased pre-heparin LPL due to omega 3 PUFA was observed.^41^

Interestingly, the weight and length of the omega 3 supplemented group were significantly higher than that of the control group after treatment. This was in agreement with the results of Collins et al.^42^ who supplemented DHA in two different doses to premature infants with less than 1250 g and more than 1250 g, and they found that infants with a birth weight of more than 1250 g had a significant increase in weight and length at 12 months of age. As regards head circumference in our study, there was no significant difference in the head circumference between both groups after treatment. In contrast to the current study, Collins et al.^42^ reported that the supplemented group with high DHA had greater head circumference than the standard control group at 12 weeks of age.

Adequate consumption of omega 3 fatty acids is vitally important during pregnancy as they are critical building blocks of the fetal brain and retina. Omega 3 fatty acids may also play a role in determining the length of gestation and in preventing perinatal depression.^43^ Additionally, based on our findings, we can theorize that omega 3 could be safe in IUGR neonates for controlling the serum leptin level and lipid profile; therefore, screening them is important to be done in all IUGR neonates at birth and during follow-up.

Nonetheless, there were certain limitations to our research. First, we did not assess important maternal information; it was reported that the mean maternal serum leptin concentration in pregnancies with IUGR was significantly higher than in normal pregnancies^44^; in addition, neonatal cord leptin concentrations correlate well with birth weight and BMI.^45^ As a result, the maternal serum leptin is an important covariate in the changes in serum leptin levels in neonates that should be considered in future studies. Second, the intervention period (2 weeks) was relatively short and could not be enough to reflect precisely the outcomes of measure. Third, we did not use a nationally representative sample in our research. As a result, before generalizing to different demographics or geographic regions, results must be carefully understood. Longer follow-up periods with a larger sample size along with long-term surveillance of all IUGR newborns for CVD detection and addressing the influence of Omega 3 on these groups are needed to better understand our findings.

## Conclusions

Omega 3 supplementations lowered serum leptin level, TG, TC, LDL, and VLDL but increased HDL and growth in neonates with IUGR.

## Data Availability

The data of this research is available from the corresponding author on reasonable request.
